# Assessing metabolic activity of yeast biofilm-forming cells on nanofibrous materials using MTT assay

**DOI:** 10.1007/s12223-025-01304-1

**Published:** 2025-07-28

**Authors:** Marta Stindlova, Vaclav Peroutka, Kamila Zdenkova, Simona Lencova

**Affiliations:** Department of Biochemistry and Microbiology, University of Chemistry and Technology, Prague, Czech Republic

**Keywords:** MTT, Metabolic activity, Yeast, Biofilm, Nanomaterials, Nanofibers

## Abstract

Nanofibrous materials (NMs), widely used in medical and food industry applications, are highly susceptible to colonisation by microorganisms, including yeasts. Although yeasts can form dense biofilms, methods for studying their metabolic activity remain limited. This study is the first to apply the MTT assay, a standardised method for assessing cell metabolic activity, to evaluate the metabolic activity of yeast biofilm-forming cells on electrospun NMs. First, the biofilm formation of *Candida albicans* and *Saccharomyces cerevisiae* on NMs electrospun from polycaprolactone (PCL), polylactic acid (PLA), and polyamide (PA) was examined. Then, key parameters of the MTT assay were systematically evaluated: (i) the addition of glucose to the MTT solution, (ii) the presence of menadione in the MTT solution, and (iii) the incubation time with the MTT solution. The addition of glucose was not proven necessary; however, in some cases, it may help distinguish the number of metabolically active cells. Based on this study, we recommend incubation with an MTT solution containing menadione for 2 h. To verify the protocol, colony-forming unit (CFU) enumeration was employed as a reference method. As differences between the results of these two methods were observed, the MTT assay should be complemented by other standardised methods. Nevertheless, the refined protocol offers a solid basis for investigating interactions between NMs and yeasts.

## Introduction

Viable and metabolically active eukaryotic cells are frequently assessed using tetrazolium salts. The main advantage of these assays is that they are relatively simple and effective. The most common salt is water-soluble 3-(4,5-dimethylthiazol-2-yl)−2,5-diphenyl tetrazolium bromide (MTT), which changes colour from a yellowish solution to water-insoluble violet formazan crystals when cleaved. However, many factors during the MTT assay must be considered for individual cell types, be it for mammalian cell lines or yeasts (Ghasemi et al. 2021).

In eukaryotic cells, tetrazolium salts are cleaved by enzymes in active mitochondria and cytoplasm (Berridge and Tan 1993). As single-cell eukaryotic microorganisms, yeasts like *Saccharomyces cerevisiae* are widely used as a research model for other eukaryotes (Vanderwaeren et al. 2022). They usually possess a cell wall consisting of β1,3-glucan fibres, sometimes chitin fibres, and the matrix of mannoproteins (Xie and Lipke 2010). Therefore, a mediator such as menadione sodium bisulphite may be needed to render yeast oxidoreductase enzyme systems more accessible during the metabolic assays (Berridge et al. 2005; Mazloum-Ardakani et al. 2019). Furthermore, it has been proven that *S. cerevisiae* reduces tetrazolium salts at only 3% of the amount compared to the human leukaemia cell line HL60 (Berridge et al. 2005).

A few studies have already focused on the modification of the MTT assay for yeasts in a liquid medium. Teparić et al. (2004) tested the survival of *S. cerevisiae* cells in yeast extract peptone dextrose medium (YPD) in 10% MTT solution. After 2-h-long incubation at laboratory temperature, acid 2-propanol was added, and the metabolic activity was measured spectrophotometrically at 540 nm. Morita et al. (2023) reported preparing yeast spheroplast cells and diluted formazan crystals in 2-propanol. Belak et al. (2018) have tested planktonic yeast cells in YPD with or without the addition of glucose and menadione, and reported the best results for the mixture of MTT with menadione and glucose solution. However, there are still many volatile factors to consider.

Furthermore, yeasts are also capable of forming biofilms that can be assessed using the MTT assay. Such biofilms consist of budding cells, hyphae, and pseudohyphae, which ensure their strength and resistance, and easily form on most materials, except highly inert ones. An example can be *Candida albicans* in the medical field and *S. cerevisiae* in the food industry (Lohse et al. 2018; Zara et al. 2020). To monitor the metabolic activity of biofilm yeast cells adherent to polystyrene (PS), Kodeš et al. (2021) have used an MTT solution in phosphate buffered saline (PBS) with 200 mmol/L glucose and an unspecified amount of menadione, proving that menadione may be an important addition in this reaction. They suggested an incubation time of 2 h and the washing solution of dimethylformamide measured at 570 nm.

Abiotic surfaces tested for yeast biofilm formation include stainless steel, wood, or glass. However, in recent decades, nanotechnology has also found broad applications in fields such as medicine, the food industry, and biotechnology. For example, nanoparticles or nanomaterials from carbon are used as either sensors or eradication measures for yeast biofilms (Alonso et al. 2023; Hosnedlova et al. 2020). Considering nanofibrous materials (NMs), many studies have investigated their role as carriers for bacterial biofilms (Hu et al. 2019; Lencova et al. 2024; Rumbo et al. 2018); nevertheless, research on yeast interactions with NMs remains limited. Most studies have focused on functionalised NMs with anti-yeast properties (Mishra et al. 2021; Sahal et al. 2019), while the fundamental interactions between non-functionalised NMs and yeasts have not yet been explored. Consequently, there is a lack of reliable protocols for studying yeast biofilms on NMs, particularly concerning their metabolic activity.

Therefore, based on our previous protocol on bacterial biofilms (Stindlova et al. 2024), we have attempted to create a reliable protocol for determining the metabolic activity of yeast biofilms on PS and NMs. We refined it and applied it to examine the metabolic activity of *S. cerevisiae* and *C. albicans* biofilms on NMs electrospun from polycaprolactone (PCL), polylactic acid (PLA), and polyamide (PA). The presented protocol can serve as a solid basis for investigating the interactions between yeasts and NMs.

## Materials and methods

### Nanomaterials

Three NMs electrospun on NS 1WS500U needle-less electrospinning device (Elmarco, Czech Republic) were tested in this study. The used polymers were PCL (Merck kGaG, Germany), PLA (Polyscitech, Indianapolis, USA), and PA (Ultramid B 27 E 01, BASG, Germany), and these NMs have already been prepared for and characterised in Stindlova et al. (2024). NMs were then sterilised by ethylene oxide (12 h, Anprolene sterilising device AN74, Andersen Sterilizer, UK) according to the ISO 11135–1 “Sterilization of products for health care sterilisation with ethylene oxide—part 1: Requirements for the development, validation and continuous control of the sterilisation procedure for medical devices” and vented at room temperature in a chemical fume hood for at least 2 weeks to ensure complete removal of residual ethylene oxide.

### Yeast strains and culture conditions

Yeasts *Candida albicans* ATCC 10231 (CA; eq. CCM 8215) and *Saccharomyces cerevisiae* ATCC 9763 (SC; eq. CCM 8191) were obtained from the Czech Collection of Microorganisms (CCM, Brno, Czech Republic). The yeasts were stored in malt extract broth (MEB, Oxoid Ltd., UK) with 25 wt% of glycerol (Penta, Czech Republic) in a freezer (− 80 °C). For recultivation, the culture was inoculated into sterile MEB and incubated for 48 h at 25 °C. For yeast suspension testing, MEB with cells of known optical density (OD) was assessed in 96-well microtiter plates (GAMA, Czech Republic) and for biofilm formation testing, MEB with yeast cell concentration of approx. 10^5^ CFU/mL was cultivated statically for 72 h at 25 °C.

### Biofilm formation

Single-species biofilms were grown from 3 mL of the suspension mentioned above in 12-well microtiter plates (TPP, Switzerland) either on the plate bottom or an NM. The 1 cm × 1 cm NMs were sterilised before each analysis by UV radiation (20 min, room temperature) and placed into the suspension. Wells without inoculum served as sterility controls (negative controls), and biofilms formed in the wells with inoculum without NMs served as positive controls. The biofilms were cultivated statically for 72 h at 25 °C.

### Determination of viable cells adhered to the nanomaterials by CFU enumeration

Viable biofilm-forming cells on the NMs were quantified according to the bacterial biofilm-focused protocol published by Lencova et al. (2021); Stindlova et al. (2024) with slight modifications for yeast cells, mainly in terms of the used media. After being washed in PBS, the biofilm-forming cells on the bottom of microtiter plates or NMs placed in microtiter plates were released into sterile MEB in a sonication bath (Polsonic, Poland; 3 min, room temperature). The obtained suspensions were decimally diluted to the sixth dilution, and droplets (20 μL) were plotted on malt extract agar (MEA, Oxoid Ltd., UK). The plates were then cultivated (72 h, 25 °C), and CFUs were counted.

### MTT assay — determination of metabolic activity of biofilm-forming cells on the nanomaterials

#### Solution preparation

All necessary solutions were prepared, handled, and stored as previously described in Stindlova et al. (2024). The tetrazolium salt stock solution was prepared by dissolving 1 mg/mL of MTT (Merck, Germany) in sterile PBS and filtering the solution through 0.22 μm filters. Glucose (Lach:ner, Czech Republic) was dissolved in sterile PBS to the concentration of 58 mg/mL. The organic solvent solution (washing solution, WS) for formazan was prepared by dissolving 160 mg/mL of sodium dodecyl sulfate (SDS; Carl Roth, Germany) in a 40% dimethyl sulfoxide (DMSO; Penta, Czech Republic) in PBS. All solutions were stored in the refrigerator and warmed up before use to either room temperature (MTT solution and glucose solution) or 45 °C (WS). Additionally, 0.2 mol/L menadione (34.4 mg/mL) was prepared and stored at − 20 °C until directly before use.

#### General layout

Yeast suspensions with OD values of 4, 2, 1, and 0.5 McFarland in MEB were inoculated into sterile 96-well PS microtiter plates (GAMA Group, Czech Republic). Biofilms on PS plates and NMs were washed three times with sterile PBS, and NMs were transferred to new sterile 12-well plates. Control NMs without yeast growth were treated similarly. With minimal light exposure, MTT solution (with or without glucose and menadione) was added to the wells without agitation, and the plates were wrapped in aluminium foil. After 2-h-long static incubation at 25 °C, the MTT solution was removed, and 1 mL of WS was added to dissolve the formazan. The plates were wrapped again and incubated statically at 37 °C for 2 h. The solutions were then mixed by pipetting five times; 100 μL of each solution was transferred to a pre-warmed sterile 96-well plate, heated, and measured spectrophotometrically at 595 nm (Tecan, Switzerland).

#### Tested conditions

Regarding our previous study, we considered the most efficient incubation time with the WS applied in our laboratory (2 h at 37 °C) and the optimal absorbance wavelength (595 nm) after heating (Stindlova et al. 2024). The tested conditions included (i) the presence of glucose in the MTT solution, (ii) the presence of menadione in the MTT solution, and (iii) the incubation time with the MTT solution. Glucose was added to the MTT solution in a ratio 7:8, and in the cases without glucose, the same ratio was obtained by adding PBS. Menadione was added to the final concentration of 0.1 mmol/L per well. The tested incubation times with respective MTT solutions were 2 h, 4 h, 6 h, 8 h, and 24 h.

#### Final protocol

1. Prepare the MTT solution in PBS, menadione solution in PBS, and washing solution (DMSO in PBS with SDS). For exact concentrations, see the “Solution preparation” section.
2. Pre-heat the washing solution to 45 °C.
3. Wash the NMs and the control wells of microtiter plates with at least 1 mL of PBS three times. Place the NMs in a new sterile microtiter plate.
4. Prepare the required amount of MTT solution in PBS in a ratio 7:8 (MTT:PBS), add the required amount of menadione (0.001 mmol/L), and mix it properly.
5. Add 600 μL of this solution to every NM and control well.
6. Avoid exposure to light and wrap the microtiter plates in aluminium foil.
7. Incubate the microtiter plates for 2 h at 25 °C to create formazan.
8. Drain the MTT solution gently and carefully but completely. Move the NMs into new sterile wells if formazan remains on the bottom of the well.
9. Add 1 mL of the pre-heated washing solution gently.
10. Incubate the microtiter plates for 2 h at 37 °C to dissolve formazan.
11. Mix the samples by pipetting five times and transfer 100 μL of every sample into the pre-warmed 96-well microtiter plate (37 °C).
12. Heat the plate for 15 min at 45 °C (with optional gentle shaking).
13. Measure the dissolved formazan spectrophotometrically at 595 nm.

### Data analysis

The experiments were performed in at least three technical and three biological replicates. For data analysis, the R programming language in RStudio was used. Data normality was verified by the Shapiro–Wilk test; the data were considered normally distributed at the significance level p > 0.05. Further, multiple comparisons by one-way analysis of variance (ANOVA) with a significance level of α = 0.05 were carried out.

## Results and discussion

The main aim of this study was to develop a robust MTT assay for evaluating the metabolic activity of yeast biofilm-forming cells on NMs. We strove for this aim by systematic steps: (i) validation of yeast biofilm-forming capability on the chosen surfaces, (ii) identification and optimisation of the critical steps in the MTT assay, (iii) validation of the optimised MTT protocol, and (iv) data analysis. The tested yeasts, *C. albicans* and *S. cerevisiae*, were selected as appropriate model organisms because they represent clinically and food-important genera which are known to form biofilms (Lohse et al. 2018; Zara et al. 2020). NMs electrospun from PCL, PLA, and PA were selected as suitable models of nanofibrous materials due to their different structures and properties, numerous applications, and widespread use in tissue engineering, medicine, food industry, and biotechnology (Aman Mohammadi et al. 2024; Cui et al. 2010; Nayl et al. 2022).

### Determination of viablecells adhered to the nanomaterials by CFU enumeration

**Fig. 1:**
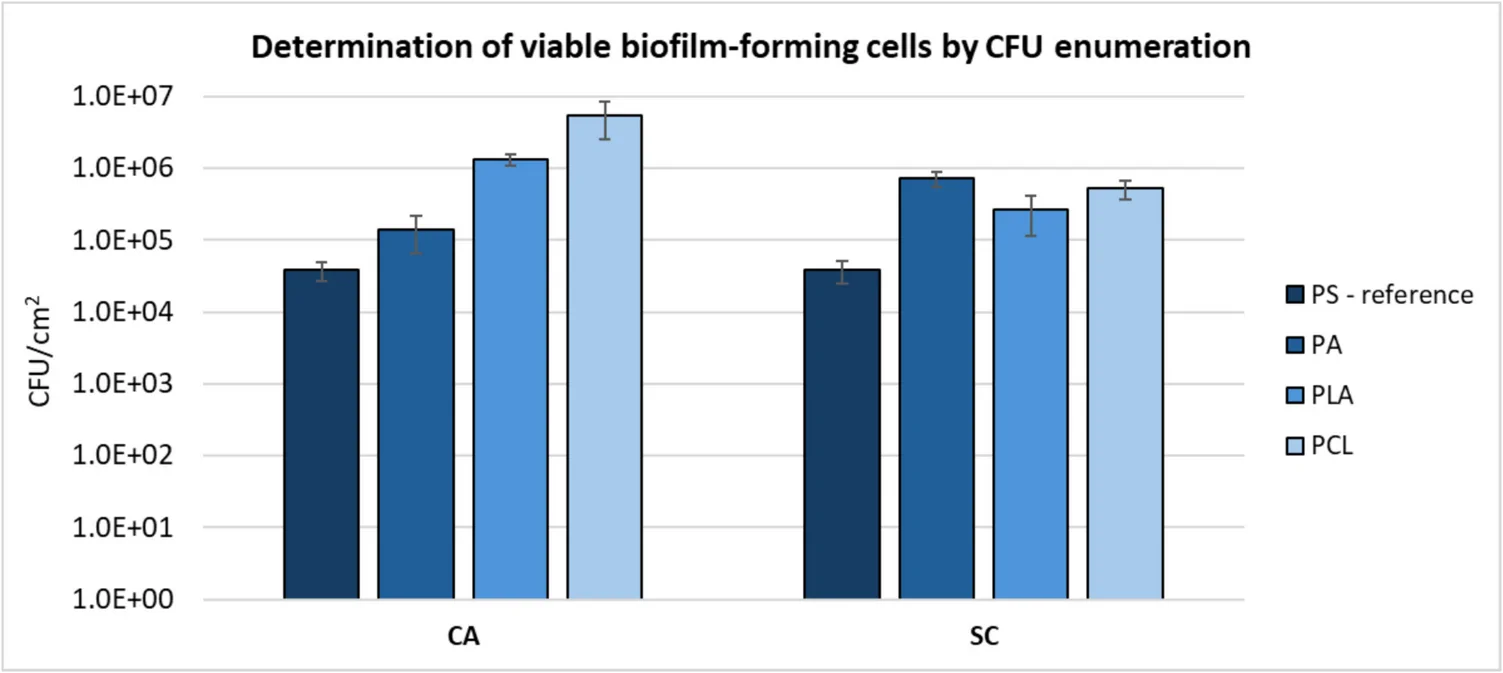
Determination of viable biofilm-forming cells of *C. albicans* ATCC 10231 (CA) and *S. cerevisiae* ATCC 9763 (SC) cultivated for 72 h on the reference material (PS), and NMs electrospun from PCL, PLA, and PA

As the first step toward a comprehensive evaluation of the MTT methodology, a quantitative analysis of viable yeast cells adhered to PS and the tested NMs was performed using the standardised colony-forming unit (CFU/cm^2^) calculation method (Fig. 1). On the reference material, PS, both yeast strains (*C. albicans* and *S. cerevisiae*) formed dense biofilms with similar cell counts. *C. albicans* biofilms exhibited an average of 3.8 ± 1.1 × 10^4^ CFU/cm^2^, while *S. cerevisiae* biofilms contained 3.8 ± 1.3 × 10^4^ CFU/cm^2^. Both yeast strains also readily colonised the tested NMs, with *C. albicans* reaching up to 5.5 ± 2.9 × 10^6^ CFU/cm^2^ and *S. cerevisiae* reaching up to 7.2 ± 1.7 × 10^5^ CFU/cm^2^. Almost all tested NMs significantly promoted biofilm formation, as the number of biofilm-forming cells was markedly higher than that on PS (p < 0.05).

Additionally, variations in biofilm formation across the three tested NMs were also observed. While the biofilms formed by *S. cerevisiae* showed relatively small variation, consistently ranging from 2.6 ± 1.4 × 10^5^ to 7.2 ± 1.7 × 10^5^ CFU/cm^2^ across all materials, *C. albicans* exhibited significantly lower colonisation of PA NMs compared to PCL and PLA (p < 0.01).

The unique properties of NMs, including a high surface-to-volume ratio, high porosity, and structures that mimic the extracellular matrix, make them highly suitable scaffolds for microbial attachment and subsequent biofilm formation (Flores-Rojas et al. 2023; Lencova et al. 2024, 2021; Rumbo et al. 2018). All three tested NMs in this study support yeast biofilm formation, which is in accordance with zein NMs by Hu et al. (2023), although different media and temperatures were used. It offers important insights into the comparison of interactions between multiple non-functionalised NMs and yeasts.

### MTT — tested conditions

Critical steps in yeast MTT assay were identified based on the published literature as follows: (i) glucose addition, (ii) menadione addition, and (iii) the incubation time with the given MTT solution. The incubation times were adjusted on planktonic cells of OD = 4, 2, 1, and 0.5 McFarland. The other steps were adapted from the bacterial MTT assay in Stindlova et al. (2024).

#### Glucose addition to the MTT solution

**Fig. 2:**
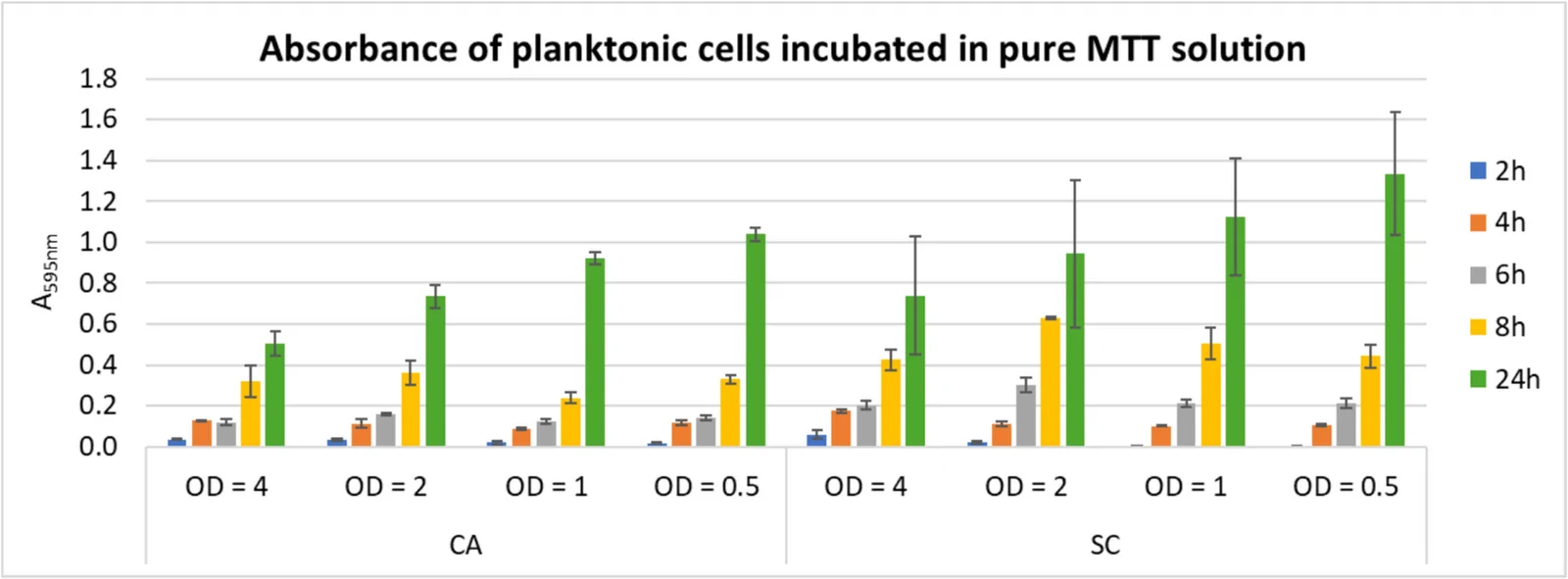
Metabolic activity of planktonic cells of *C. albicans* ATCC 10231 (CA) and *S. cerevisiae* ATCC 9763 (SC) represented by absorbance at 595 nm (mean ± SD) after different periods of incubation with pure MTT

First, we tested all chosen incubation times (2, 4, 6, 8, 24 h) on planktonic yeast cells in the pure MTT solution to assess how much yeasts can metabolise MTT without additional compounds (Fig. 2). The results showed that incubation in pure MTT solution could not well distinguish between different OD, starting from 4 h of incubation. Furthermore, prolonged incubation of 24 h resulted in higher standard deviations in *S. cerevisiae* and higher absorbance in the lowest concentration of both yeast planktonic cells.

**Fig. 3:**
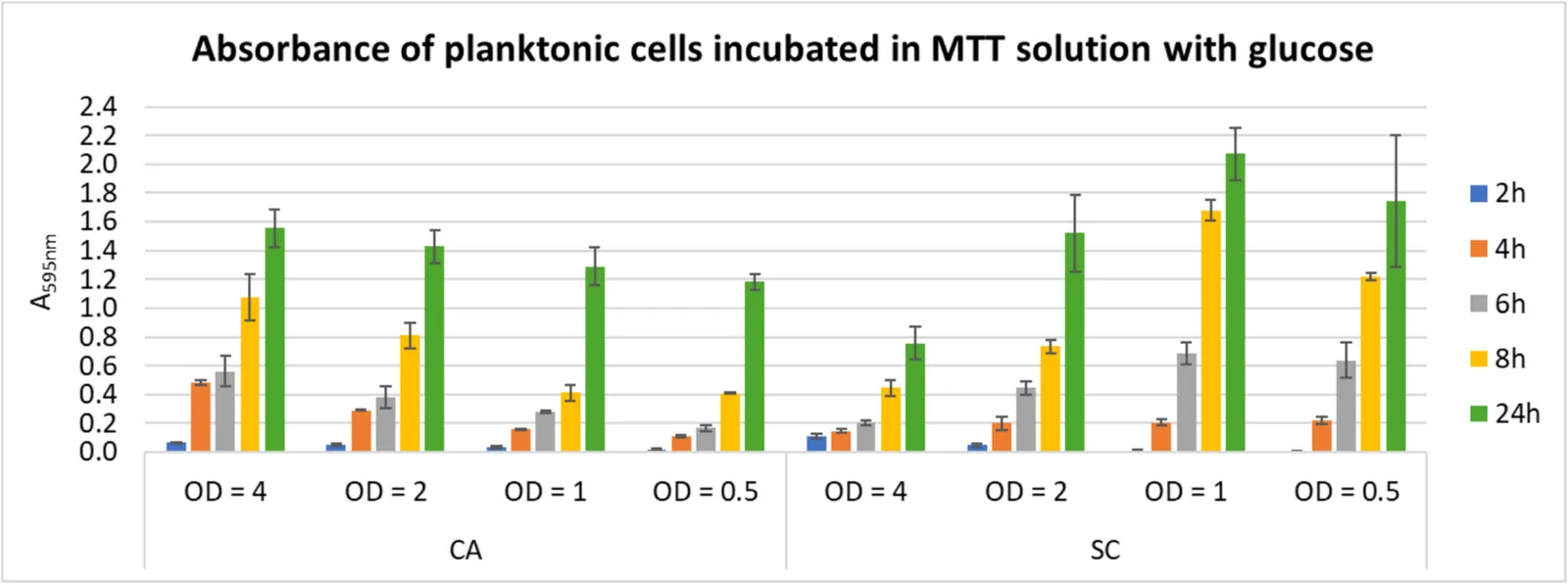
Metabolic activity of planktonic cells of *C. albicans* ATCC 10231 (CA) and *S. cerevisiae* ATCC 9763 (SC) represented by absorbance at 595 nm (mean ± SD) after different periods of incubation with MTT solution supplied with glucose

Therefore, similarly to the bacterial MTT assay, we added glucose to the solution and tested all four yeast concentrations at the five chosen time points (see Fig. 3). Glucose addition could better distinguish the number of metabolically active cells in the case of *C. albicans* after 4 h and more. However, this was not the case for *S. cerevisiae*, where longer incubation time resulted in higher absorbance values at lower OD. These findings foreshadow the differences in MTT metabolism in the presence of glucose between these two yeast species. Moreover, as the reduction of MTT by mitochondrial enzymes is directly proportional to the cell metabolic activity, in the cases of low activity, a higher number of cells is required to produce detectable levels of MTT reduction at a shorter time (Levitz and Diamond 1985).

#### Menadione addition to the MTT solution

**Fig. 4:**
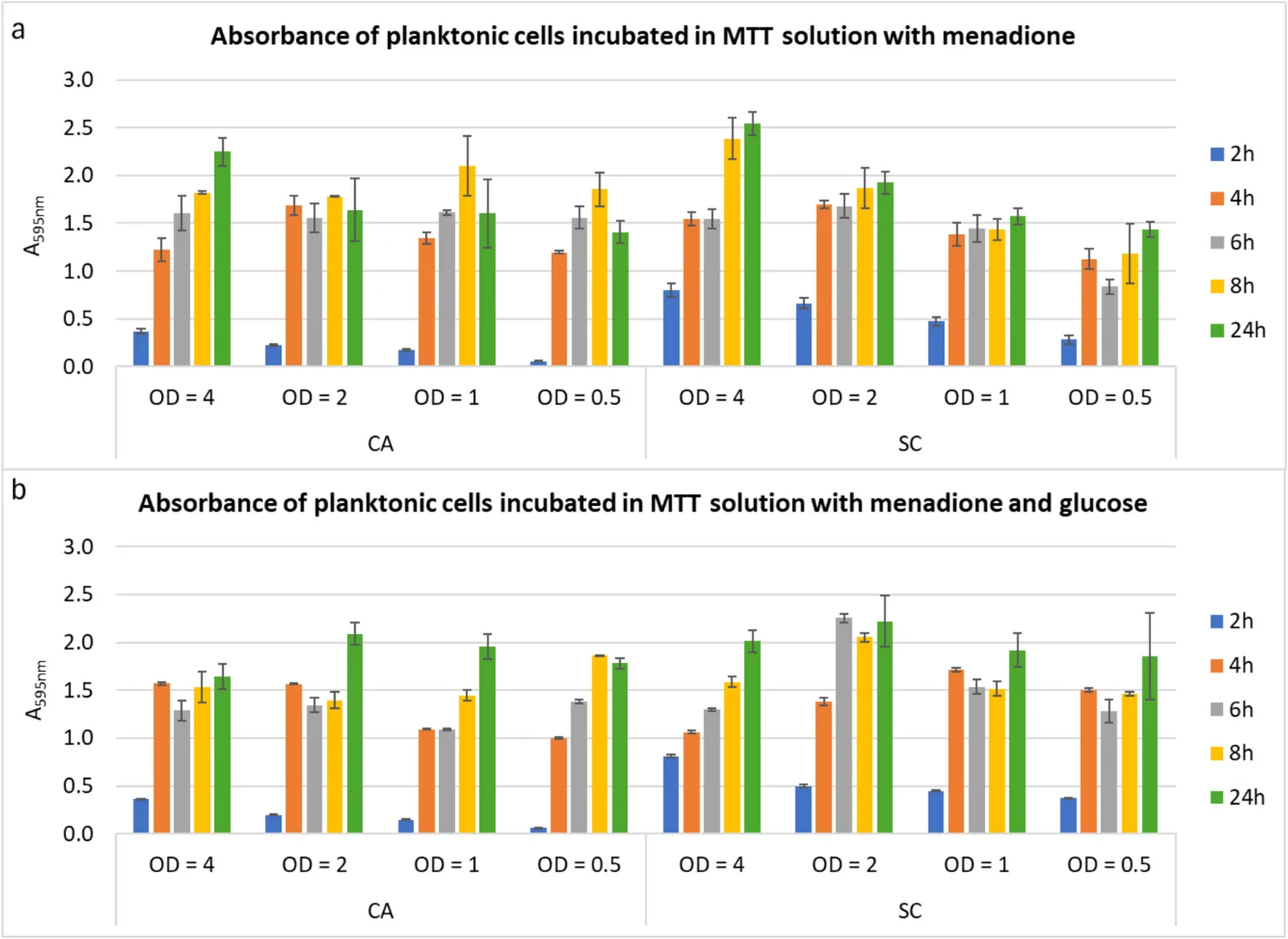
Metabolic activity of planktonic cells of *C. albicans* ATCC 10231 (CA) and *S. cerevisiae* ATCC 9763 (SC) represented by absorbance at 595 nm (mean ± SD) after different periods of incubation with MTT solution supplied with menadione (**a**) or menadione and glucose (**b**)

To address the assay limitations for cells with slow MTT metabolism, lipophilic menadione was introduced in the 1990s. It facilitates electron transfer from intracellular redox systems through the cell membrane to the extracellular environment, where the reduction of the dyes occurs (Garn et al. 1994). Therefore, to make the assay more robust and applicable to both yeast species, we also measured the absorbance of planktonic cells incubated for these five periods of time with the addition of menadione both without (Fig. 4a) and with (Fig. 4b) glucose like in the study of Belak et al. (2018).

The addition of menadione greatly enhanced the absorbance for both yeast species at all chosen times, and it even proved suitable for the shortest period of incubation with the MTT solution. Moreover, the effect of menadione was not influenced by the addition of glucose but it gave better yields than glucose on its own. Therefore, the most suitable combination of time and supplement appeared to be 2 h with only the menadione addition.

Our results correlate with the study of Belak et al. (2018), who found that the MTT assay using glucose and menadione could more effectively detect the metabolic activity of *S. cerevisiae*. However, menadione (0.2 mmol/L) supplemented with glucose had the highest absorbance rate and was well detectable as early as after 2 h, with a gradual non-linear increase of absorbance over time. Similar trends can be observed in some of our datasets, but the effect of MEB also plays an important part in the absorbance rate because yeasts could proliferate slowly.

On the other hand, although Ansari et al. (2016) successfully analysed *C. albicans* cell suspensions using an MTT assay without menadione, their cell concentration and cultivation temperature were higher than in our study (> 3 × 10^6^ CFU/mL at 30 °C). Other studies use the menadione-supplied XTT assay. It is more sensitive than MTT, provides consistent results for *Candida* biofilms, and the orange formazan is measured directly at 490 nm. However, it is advised to supplement it with crystal violet staining (Dogan et al. 2021; Ferreira et al. 2013; Zhu et al. 2013). Therefore, it could be easier for laboratories to measure crystal violet and MTT assay using the same wavelength, namely 595 nm, still keeping in mind that crystal violet may bind into NM structure (depending on the polymer used) and thus bias the results (Lencova et al. 2021).

Based on our results and the mentioned studies, we decided to further investigate biofilm metabolic activity after incubation for 2 h and 4 h in MTT with menadione.

### Assay application to biofilm on polystyrene

**Fig. 5:**
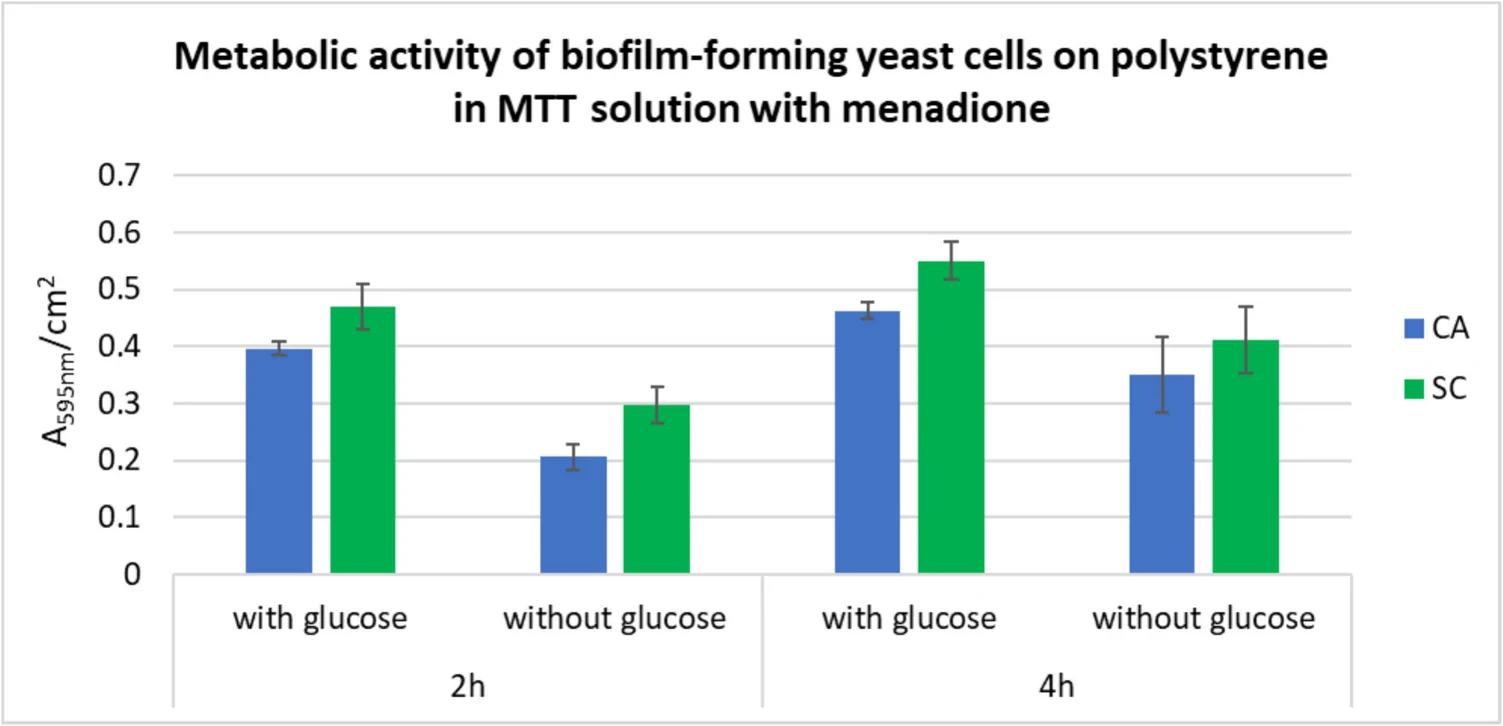
Metabolic activity of biofilms of *C. albicans* ATCC 10231 (CA) and *S. cerevisiae* ATCC 9763 (SC) on polystyrene microtiter plates represented as absorbance at 595 nm (mean ± SD). The biofilms were incubated in an MTT solution supplemented with menadione with or without glucose for 2 h and 4 h

First, we tested the yeast biofilm formed on PS as the reference material, considering the time consumption and simplicity of the assay. As yeast cells in biofilms are less sensitive to outer stimuli than planktonic cells (Zara et al. 2020), we observed the two shortest incubation times in MTT with menadione both with and without glucose addition (Fig. 5).

For both yeasts, incubation for 4 h yielded significantly higher absorbance (p < 0.05) than for 2 h regardless of the glucose addition. However, if compared within individual time points, the glucose addition significantly raised absorbance at 595 nm (p < 0.05) — see Fig. 5. In recent studies like Kodeš et al. (2021), glucose is added empirically into the MTT solution with menadione, although similar representative results could be obtained without glucose at a slightly longer incubation time of 4 h (Garn et al. 1994; Imbert et al. 2002). Our results suggest that even 2 h without glucose addition could still provide high enough absorbance under the tested conditions. Considering the time factor and the possibility of aligning the yeast MTT protocol with the bacterial one, the period of 2 h was chosen for the final version of the proposed protocol.

### Final assay application to nanomaterials

**Fig. 6:**
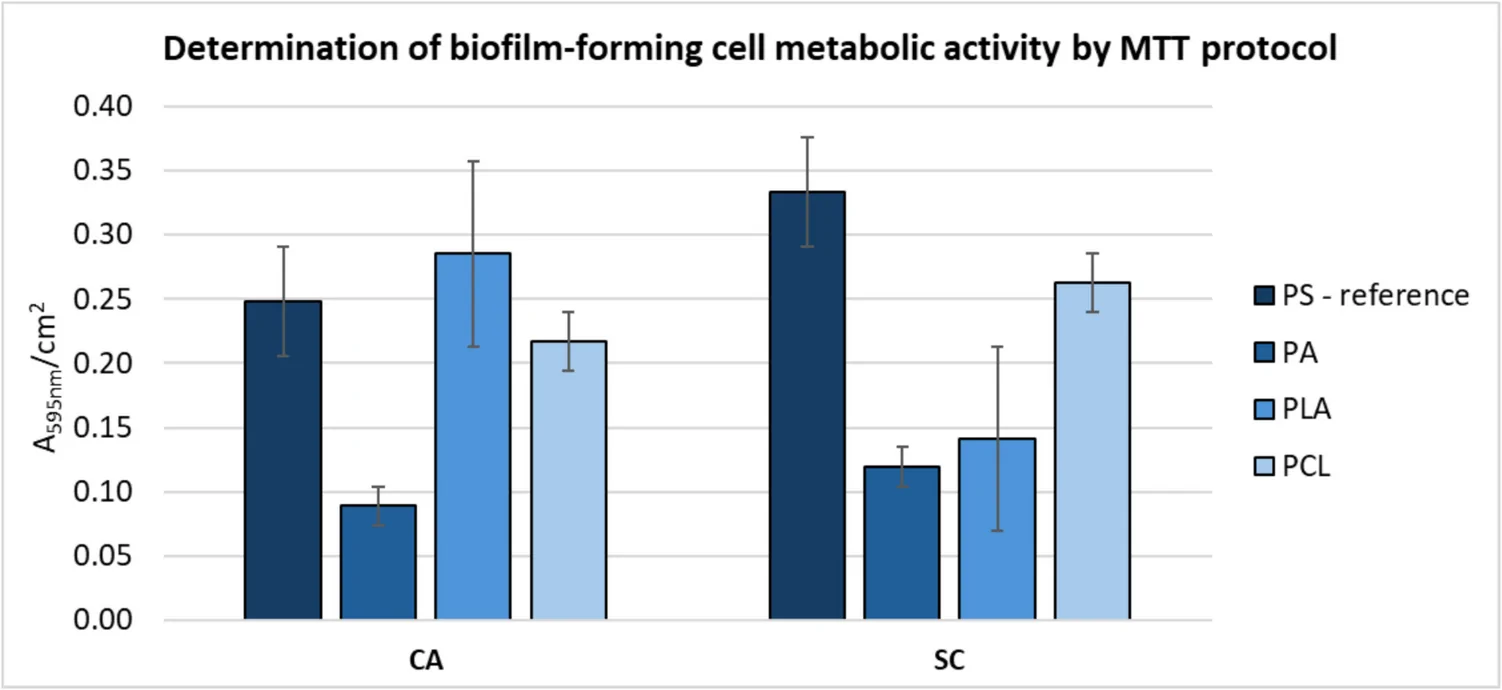
Determination of biofilm-forming cell of *C. albicans* ATCC 10231 (CA) and *S. cerevisiae* ATCC 9763 (SC) on the bottom of a microtiter plate (polystyrene, PS — reference) and NMs (PCL, PLA, and PA) represented as absorbance at 595 nm (mean ± SD)

Subsequently, we applied the proposed protocol (2-h-long incubation in the MTT solution with menadione but without glucose) to test the metabolic activity of biofilm-forming cells of *C. albicans* and *S. cerevisiae* on PA, PLA, and PCL NMs and PS (Fig. 6). We have successfully determined the metabolic activity of biofilm-forming cells; however, the trends were vastly different from the CFU enumeration (Fig. 1).

In the case of *C. albicans*, PS seemed to be the least suitable material for biofilm formation, but the metabolic activity of these cells was comparable to that of biofilms formed on PLA and PCL NMs. The only significantly lower metabolic activity was found on PA (p < 0.01). In the case of *S. cerevisiae*, metabolic activity on all three tested NMs was lower than on PS (not significantly for PCL, p > 0.05) despite the significantly higher CFU count. These results highlight differences in biofilm-forming cell metabolism on the tested materials and a necessity for further study of yeast growth on NMs as Stepanenko and Dmitrenko (2015) have concluded that growth conditions can greatly affect the accuracy of the MTT assay. Despite that, it seems that cells on PA are the least metabolically active for both species.

The MTT assay has been proven to detect subtle changes in metabolic activity caused by various treatments, and even lower biofilm mass can produce as much formazan as the untreated control (Kodeš et al. 2021). However, CFU enumeration does not consider viable but nonculturable cells (VBNC), which are still metabolically active but cannot be revived easily by plating. Yeast VBNC receive even less attention than bacterial ones due to the lack of a clear understanding of this state in eukaryotes (Xiao et al. 2025). Both *C. albicans* and *S. cerevisiae* can turn into VBNC in the presence of a stressor, as proven by Salma et al. (2013) for *S. cerevisiae* or Xie et al. (2021) for *C. albicans*. Another means of discrepancies between the two methods is the accessibility of cells to MTT in NMs because cells can not only cover the material in a uniform layer but also grow in the spaces between fibres. Furthermore, the diffusion of formazan from individual NM can vastly differ based on the polymer used. Therefore, if applied, it is necessary to have an untreated control for every tested NM to assess how much metabolic activity can be measured and compare the results of treated samples to this value.

In summary, we developed a robust protocol (see the “Final protocol” section) for determining the metabolic activity of biofilm-forming yeast cells on NMs electrospun from PA, PLA, and PCL. The protocol’s parameters, including NM size of 1 cm^2^, reagent volumes, and incubation conditions, are optimised for use with *C. albicans* and *S. cerevisiae* in a 12-well microtiter plate format. These parameters should be validated when applying the protocol to different materials or organisms; we especially recommend considering the specific characteristics of the tested microorganisms and adjusting the protocol as necessary for the particular application. This protocol can be employed as a complementary method alongside CFU enumeration or other techniques to assess the total number of metabolically active cells.

## Conclusion

The MTT assay is widely employed to assess the metabolic activity of both prokaryotic and eukaryotic cells. In this study, it was applied for the first time to evaluate the metabolic activity of biofilm-forming *C. albicans* and *S. cerevisiae* cells within biofilms on nanofibrous materials composed of PA, PLA, and PCL, respectively. Various experimental conditions were tested, and based on the results, we recommend performing the assay with the addition of menadione, excluding glucose, and incubating the MTT solution for 2 h. This protocol can be utilised to assess yeast-nanomaterial interactions; however, before application, it is crucial to consider the specific growth conditions of the different yeast species and the properties of the polymers used to fabricate the nanofibers.

## Data Availability

Data will be made available on request.

## Abbreviations

*CA*: *C. albicans* ATCC 10231
*CCM*: The Czech Collection of Microorganisms
*CFU*: Colony-forming units
*DMSO*: Dimethyl sulfoxide
*MEA*: Malt extract agar
*MEB*: Malt extract broth
*MTT*: 3-(4,5-Dimethylthiazol-2-yl)-2,5-diphenyl tetrazolium bromide
*NM*: Nanofibrous material
*OD*: Optical density
*PA*: Polyamide
*PBS*: Phosphate buffered saline
*PCL*: Polycaprolactone
*PLA*: Polylactic acid
*PS*: Polystyrene
*SC*: *S. cerevisiae* ATCC 9763
*SDS*: Sodium dodecyl sulfate
*VBNC*: Viable but nonculturable cells
*WS*: Washing solution
*YPD*: Yeast extract peptone dextrose medium
